# Effect of Aqueous Extract of *Adansonia digitata* Stem Bark on the Development of Hypertension in L-NAME-Induced Hypertensive Rat Model

**DOI:** 10.1155/2020/3678469

**Published:** 2020-09-18

**Authors:** Fidèle Ntchapda, Christian Bonabe, Albert Donatien Atsamo, David Romain Kemeta Azambou, Yannick Bekono Fouda, Soudy Imar Djibrine, Paul F. Seke Etet, Dimo Théophile

**Affiliations:** ^1^Department of Biological Sciences, Faculty of Sciences, University of Ngaoundéré, P.O. Box 454, Ngaoundéré, Cameroon; ^2^Department of Animal Biology and Physiology, Laboratory of Animal Physiology, University of Yaoundé I, P.O. Box 812, Yaoundé, Cameroon; ^3^Institut Universitaire des Sciences et Techniques d'Abéché (IUSTA), P.O. Box 6077, N'Djamena, Chad; ^4^Department of Physiological Sciences and Biochemistry, FMBS, University of Ngaoundéré, Ngaoundéré, P.O. Box 454, Cameroon

## Abstract

**Background:**

*Adansonia digitata* is a plant used against cardiovascular disorders in African folk medicine. We assessed the effects of the aqueous extract of its stem bark on the development of hypertension in L-NAME-induced hypertensive rats.

**Methods:**

The animals were administered L-NAME once daily for 3 weeks (25 mg/kg, i.p.), concomitantly with aqueous extract of *A. digitata* stem bark (100 and 200 mg/kg, p.o.) or captopril (20 mg/kg, p.o.). Then, hemodynamic and electrocardiographic parameters, oxidative stress markers, and the lipid profile were assessed in the blood and heart, aorta, and kidney homogenates, and histopathological analyses were performed.

**Results:**

L-NAME-induced hypertensive control animals, but not the animals concomitantly treated with *A. digitata* extract, displayed increases in the mean arterial blood pressure (21.64% difference, *p* < 0.001, vs. dose 200 mg/kg), systolic arterial blood pressure (21.33%, *p* < 0.001), and the diastolic arterial blood pressure (21.84%, *p* < 0.001). In addition, hypertensive control animals displayed (i) increases in serum triglycerides, total cholesterol, LDL, and creatinine levels, malondialdehyde and transaminase activities, and atherogenic index; (ii) decreases in serum HDL, catalase, reduced glutathione, and nitric oxide; and (iii) aorta wall thickening, inflammatory cell infiltration, and cell loss in the cardiac muscle and renal tissues. As captopril, the extract prevented hypertension-like changes in lipid profile, cardiac, hepatic, and renal affection indicators, and oxidative stress markers.

**Conclusion:**

Our findings suggest that the extract of *A. digitata* has antihypertensive and antioxidant effects in L-NAME-induced hypertension rat models. These effects partly justify the traditional medicine use against cardiovascular disorders.

## 1. Introduction

High blood pressure (hypertension) is a major driver of cardiovascular diseases which can lead to life-threatening conditions such as myocardial infarction, coronary heart failure, renal failure, and stroke [1, 2]. The incidence of hypertension has been increasing worldwide, particularly in developing countries. For instance, from 80 million adults in the early 2000s, the number of hypertension cases in sub-Saharan Africa is expected to rise to 150 million by 2025 [3, 4]. The pathogenetic mechanisms of hypertension encompass complex interactions of signaling pathways and genetic and environmental factors.

Nonetheless, it is well-established that oxidative stress contributes to the development of hypertension through nitric oxide (NO) deficiency [5, 6]. The vascular endothelial cells' product NO is a potent vasodilator with important roles in the growth and resistance of blood vessels [7, 8]. NO synthase inhibitors induce endothelial dysfunction and oxidative stress by decreasing NO activity. Subchronic administration of laboratory rodents with NO synthase inhibitors, such as N-nitro-L-arginine methyl ester (L-NAME), results in chronic hypertension [9, 10], hence the common use of this chemical for developing hypertension experimental models.

Beyond the pathogenesis, the management of hypertension without marked undesired effects and consequently with high patient adherence is challenging [5–8]. Novel therapeutics are direly needed in the field, and medicinal plants are a potential source. *Adansonia digitata* L. (“African baobab”) is a medicinal plant of the order of Malvales (the Bombaceae family), which is commonly used by African traditional healers for the management of cardiovascular disorders [11]. *A. digitata* is a big iconic and culturally significant tree found in savannahs of sub-Saharan Africa, which is popularly nicknamed as “the small pharmacy tree” due to its numerous uses in folk medicine [12, 13]. The plant was reported anti-inflammatory, antioxidant, antimalarial, and antimicrobial properties [14, 15]. The stem bark contains a semifluid gum that is used in folk medicine to clean wounds, to promote weight gain, and as growth food for infants [16]. An alkaloid isolated from the stem bark and termed as “adansonine” was reported to account at least partly for antimalarial [17] and antidepressant properties [18, 19]. The fruits of *A. digitata* were reported cardioprotective properties against isoproterenol-induced cardiac damage [20, 21]. However, despite the traditional use against cardiovascular diseases, data are lacking on the effects of the plant on hypertension, a major driver of these diseases.

In the present study, we assessed the effect of the aqueous extract of *A. digitata* stem bark on the development of hypertension in L-NAME-induced hypertensive rats.

## 2. Material and Methods

### 2.1. Animal Material

Adult male Wistar albino rats (*N* = 30, about 9-weeks old, 150–160 g) were obtained from the animal facility of the Faculty of Science (FS), University of Ngaoundéré. They were housed under 25 ± 1°C room temperature, 43 ± 10% relative humidity, and 12 : 12°h light/dark cycle, in a room of the Laboratory of Medicinal Plants, Health and Galenic Formulation, Department of Biological Sciences (FS), University of Ngaoundéré. The animals had free access to standard diet and tap water.

The experimental protocol was approved by the Institutional Animal Ethics Committee (reference no. FWIRB 00001954). Research activities were conducted following European Community guidelines for laboratory animal use and care (86/609/EEC).

### 2.2. Experimental Design

The rats were randomly divided into five groups (*N* = 6 per group). After one week of acclimation to laboratory conditions, for three consecutive weeks and once daily, the animals were administered with either physiological solution (NaCl 0.9% in a volume of 0.5 ml/100 g, i.p.) (the normotensive control group) or L-NAME (25 mg/kg in a volume of 0.5 ml/100 g, i.p.) (hypertensive-like groups). The latter groups concomitantly received either the physiological solution in a volume of 0.5 ml/100 g (i.p.) (the hypertensive control group), the antihypertensive drug captopril (20 mg/kg, in a volume of 1 mL/100 g, p.o.) (the positive control group), or a dose of the aqueous extract of *A. digitata* (100 or 200 mg/kg in a volume of 1 mL/100 g, p.o.) (test groups). The body weight was measured every two days.

At the end of the 3 weeks of L-NAME daily administration, the animals were anesthetized and the femoral artery exposed by dissection to measure the blood pressure and the heart rate. Then, the arteriovenous blood was collected in heparinized tubes and animals were sacrificed. The aorta, kidney, and heart were dissected out. Fats and other connective tissues were removed, and organs were weighed. Samples were processed for biochemical tests and histopathological studies.

### 2.3. Plant Extract Preparation

The stem bark of *A. digitata* was harvested in the town of Maroua, Diamaré, Far North Region (Cameroon), in November 2017. A sample was identified, authenticated, and stored (Cameroon National Herbarium Specimen No 42417/HNC, Yaoundé). Stem bark peels were air-dried at room temperature, grounded to powder using an electric blender, and stored in a glass container. About 100 g of the powder was macerated in 1000 mL of distilled water for 24 hours. The solution was filtered using Wattman No. 3 paper. Then, the filtrate was evaporated using an oven (45°C). The solid extract obtained (yield: 31.4%) was stored at 4°C. Every day, the solid extract was used to prepare fresh solutions for the oral administration of the doses 100 and 200 mg/kg of extract in a volume of 1 mL/100 g (p.o.).

### 2.4. Hemodynamic and Electrocardiographic Measurements

The animals were deeply anesthetized (urethane, 1.5 g/kg, i.p.). The trachea was exposed and cannulated to ensure normal respiration, while the femoral artery was exposed, cannulated, and flushed with 1 ml of heparinized Mc Even buffer. Then, the blood pressure and the heart rate were measured by connecting the cannulated femoral artery to a computerized system including an arterial cannula connected to a pressure transducer coupled with a hemodynamic recorder (MP35, Biopac Student Lab, Goleta, CA, USA).

### 2.5. Biochemical Tests

#### 2.5.1. Blood Samples

The arteriovenous blood collected was centrifuged (3000 rpm, 15 min, 4°C), and the plasma was separated and used for biochemical analyses. Triglycerides and LDL-, HDL-, and total cholesterol were determined using GIESSE kit, according to the instructions of the manufacturer (GIESSE Diagnostics, Roma, Italy). Standard biochemical methods were used to measure plasma levels of proteins, transaminases, urea, and creatinine [22–24]. The atherogenic index (AI) was calculated as follows:

AI°=°([total cholesterol] − [HDL − cholesterol])/[HDL − cholesterol] [25].

#### 2.5.2. Organ Homogenates

Samples of the aorta, kidney, and heart tissues were homogenized (20%°w/v) in Tris-HCl buffer (kidney) or in Mc Even buffer (heart and aorta). The homogenates obtained were centrifuged (4000 rpm, 25 min, 4°C). The supernatant was collected and processed for the analysis of antioxidant enzyme activities. Standard biochemical techniques were used to measure the activities of reduced glutathione (GSH) [26] and catalase [27], the nitrite content [28], and the malondialdehyde concentration [29].

### 2.6. Histopathological Analyses

Samples of the aorta, the heart, and kidney were fixed in 10% buffered formaldehyde and processed for paraffin embedding. Then, they were cut using a microtome (section thickness: 4 *μ*m), and sections were stained using H&E. Histopathological changes were characterized by analyzing the sections stained under a computerized microscope equipped with a camera (Olympus, Hamburg, Germany).

### 2.7. Statistical Analysis

The results were analyzed using Microsoft Excel 2019 and GraphPad Prism software (version 7.0). Data obtained in the test groups and in the positive control group were compared with those obtained in the normotensive control group and in the hypertensive control group. The statistical significance of differences was assessed using one-way ANOVA followed by Tukey *post hoc* test for intergroup comparisons. Differences with *p* value < 0.05 were significant. Data were presented as mean ± SEM.

## 3. Results

### 3.1. Body Weight and Cardiovascular Properties

#### 3.1.1. Body and Organ Weights

Table 1 presents the changes in body weight before and after 3 weeks of concomitant treatment with L-NAME and either the aqueous extract of *A. digitata* or captopril. A marked decrease in body weight growth was observed in the hypertensive control group (Table 1). The aqueous extract at all doses tested and captopril mitigated this decrease. Gains of 23.36% (*p* < 0.05), 94.13% (*p* < 0.001), and 27.02% (*p* < 0.05) were observed with doses 100 and 200 mg/kg, and captopril, respectively, was compared to the hypertensive control group (Table 1).

Table 1 also shows the effects of concomitant treatment with L-NAME and either the aqueous extract of *A. digitata* or captopril on the relative weight of the heart, the left ventricle, and kidneys. The relative weights of the heart and of left ventricle were increased significantly (27.67 %, *p* < 0.05, and 48.07%, *p* < 0.001, respectively) in hypertensive control animals (Table 1). Treatment with captopril and plant extract doses 100 and 200 mg/kg mitigated this increase (Table 1). Relative weight of kidneys was also increased significantly in the hypertensive control group (0.79 ± 0.05 vs. 0.59 ± 0.05 g/100 g BW in the normotensive control group, *p* < 0.05) (Table 1). This detrimental change was prevented in groups receiving the extract at doses 100 or 200 mg/kg, or captopril (*p* < 0.05) (Table 1).

#### 3.1.2. Blood Pressure and Heart Rate

Table 2 presents the systolic arterial blood pressure (SBP), the diastolic arterial blood pressure (DBP), the mean arterial blood pressure (MABP), and the heart rate of animals treated daily and concomitantly with L-NAME and either the aqueous extract of *A. digitata* or captopril for 3 weeks. The SBP, DBP, and MBP were markedly increased in the hypertensive control group compared to the normotensive control group (*p* < 0.001) (Table 2). Treatment with the aqueous extract of *A. digitata* at doses 100 and 200 mg/kg and with captopril mitigated this change (*p* < 0.001 vs. the hypertensive control group and *p* < 0.05 vs. normotensive controls) with differences in SBP, DBP, and MBP of (Table 2): (i) 8.89 %, 6.44%, and 8.23% with extract dose 100 mg/kg, respectively (compared to the hypertensive control group); (ii) 21.33%, 21.84%, and 21.64% with extract dose 200 mg/kg, respectively; and (iii) 22.33%, 19.43%, and 20.45% with captopril, respectively.

The heart rate was also markedly increased in the hypertensive control group compared to the normotensive control group (*p* < 0.001) (Table 2). Treatment with the aqueous extract of *A. digitata* at doses 100 and 200 mg/kg and with captopril prevented this change and maintained the heart rate close to normotensive control values (Table 2).

#### 3.1.3. Electrocardiographic Parameters

Table 3 presents the electrocardiographic parameters of animals treated daily and concomitantly with L-NAME and either the aqueous extract of *A. digitata* or captopril for 3 weeks. Hypertensive control group, but not animals treated with the aqueous extract of A. digitata (100 and 200 mg/kg) or captopril, displayed marked increases in QRS interval duration, thus increasing the magnitude (Table 3). In addition, the administration of the aqueous extract of *A. digitata* significantly prevented the decrease in the duration of the PR interval observed in the hypertensive control animals with 42.67 ± 0.29 ms in the group treated with the extract at dose 100 mg/kg, and 49.33 ± 0.58 ms in the group treated with extract dose 200 mg/kg, against 48.33 ± 0.76 ms in the hypertensive control group (i.e., decrease of 15.60% and 13.26%, respectively, *p* < 0.001) (Table 3).

Unlike at 100 mg/kg, at 200 mg/kg the aqueous extract of *A. digitata* significantly prevented the reduction in the duration of the QRS complex observed in hypertensive control animals (44.30%, *p* < 0.05) (Table 3). Both the doses of *A. digitata* extract (100 and 200 mg/kg) and captopril prevented significantly the increase in the duration of the QT interval observed in the hypertensive control animals (65.3 ± 1.0, 65.6 ± 1.5, and 62.0 ± 0.3, respectively, vs. 85.33 ± 3.97, *p* < 0.01) (Table 3). Also compared to hypertensive control animals, slight increases were observed in the RR interval duration of animals treated with the extract (169.67 ± 0.76 with dose 100 mg/kg and 156.00 ± 1.00 ms with dose 200 mg/kg vs. 151.33 ± 0.76 ms in the hypertensive control group) (Table 3).

### 3.2. Oxidative Stress Markers and Lipid Profile

#### 3.2.1. Nitric Oxide and Malondialdehyde Levels

**Figure 1:**
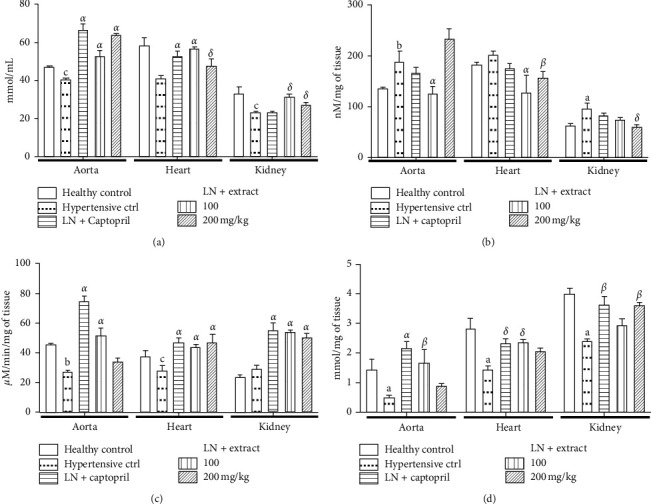
Oxidative stress indicators. Effects of the aqueous extract of *Adansonia digitata* on aorta, heart, and kidney levels or activities of (a) nitrite, (b) malondialdehyde, (c) catalase, and (d) reduced glutathione.

Figures 1(a) and 1(b) show the changes in nitric oxide (Figure 1(a)) and malondialdehyde (Figure 1(b)) levels of animals treated daily and concomitantly with L-NAME and either the aqueous extract of *A. digitata* or captopril for 3 weeks. Compared to normotensive control animals, the hypertensive control group displayed significant decreases in levels of nitric oxide in the aorta (18.80%, *p* < 0.05), in the heart (30.95%, *p* < 0.001), and in the kidney (29.73%, *p* < 0.05) tissues (Figure 1(a)). Treatments with extract doses 100 and 200 mg/kg mitigated significantly the decreases in nitric oxide in the aorta (differences compared with the hypertensive control group: 30.30%, *p* < 0.01, and 57.39%, *p* < 0.001, respectively), in the heart (40.39%, *p* < 0.001, and 18.10%, not significant, respectively), and in the kidney (198.78%, *p* < 0.001, and 114.29%, *p* < 0.001, respectively) (Figure 1(a)). Captopril also mitigated significantly the decreases in nitric concentration in the aorta (64.53%, *p* < 0.001), in the heart (30.54%, *p* < 0.05), and in the kidney (16.72%, *p* < 0.05) compared to the hypertensive control (A).

Bars are mean ± SEM (*N* = 6 per group). ANOVA + the Tukey test: ^c^*p* < 0.05, ^b^*p* < 0.01, and ^a^*p* < 0.001 vs. the normotensive control group; ^*δ*^*p* < 0.05, ^*β*^*p* < 0.01, and ^*α*^*p* < 0.001 vs. the diabetic control group. LN = N-nitro-L-arginine methyl ester.

As shown in Figure 1(b), hypertensive control animals displayed significant increases in malondialdehyde levels in the aorta (53.48%, *p* < 0.01) and in the kidney (39.12%, *p* < 0.05), but not in the heart, as compared to the normotensive control group (Figure 1(b)). Compared to the hypertensive control animals, the rats concomitantly treated with L-NAME and dose 200 mg/kg of *A. digitata* extract displayed significantly lower levels of malondialdehyde in the heart (37.22%, *p* < 0.01) and in the kidney (22.38%, *p* < 0.05) (Figure 1(b)). Also compared to the hypertensive control animals, the rats treated with extract dose 100 mg/kg displayed significantly lower malondialdehyde levels (*p* < 0.001) in the aorta (32.43% difference) and in the heart (36.29%) (Figure 1(b)). Captopril did not induce any marked change in malondialdehyde tissue levels.

#### 3.2.2. Catalase Activity and Reduced Glutathione Level

Figures 1(c) and 1(d) show the changes in reduced glutathione level (Figure 1(c)) and catalase activity (Figure 1(d)) levels of animals treated daily and concomitantly with L-NAME and either the aqueous extract of *A. digitata* or captopril for 3 weeks. Catalase activity was decreased in the aorta and in the heart of hypertensive control animals compared to the normotensive control group (Figure 1(c)). *A. digitata* (100 and 200 mg/kg) treatment increased significantly the catalase activity in the aorta (68.68%, *p* < 0.01, and 75.68%, *p* < 0.001, respectively) and in the kidney (92.25%, *p* < 0.001, and 57.30%, *p* < 0.01) (Figure 1(c)). Treatment with captopril significantly increased catalase activity in the heart (86.64%, *p* < 0.001) and in the kidney (88.36%, *p* < 0.001) compared to the hypertensive control group (Figure 1(c)).

Hypertensive control animals displayed significant decreases in the glutathione levels in the aorta (29.51%, *p* < 0.001), in the heart (34.46%, *p* < 0.001), and in the kidney (29.11%, *p* < 0.001) compared to the normotensive control group (Figure 1(d)). The animals treated with the extract dose 200 mg/kg significantly mitigated this decrease in the kidney (27.14%, *p* < 0.01, compared to hypertensive rats), while the animals treated with the dose 100 mg/kg mitigated the decrease in the aorta (*p* < 0.01) and in the heart (*p* < 0.001) (Figure 1(d)). Captopril treatment also mitigated the decrease in reduced glutathione levels in the aorta (106.60%, *p* < 0.001) and in the kidney (28.07%, *p* < 0.01) (Figure 1(d)).

#### 3.2.3. Blood Lipid Profile

Table 4 presents the blood lipid profile of animals treated daily and concomitantly with L-NAME and either the aqueous extract of *A. digitata* or captopril for 3 weeks. The hypertensive control animals displayed significant (*p* < 0.05) increases in blood total cholesterol (23.31%), triglycerides (53.61%), LDL-cholesterol (61.31%), and decrease in HDL-cholesterol (63.04%) compared to the normotensive control group (Table 4). Extract dose 200 mg/kg significantly (*p* < 0.05) prevented the increases in total cholesterol (26.34% difference from the hypertensive control group) and in triglycerides (25.64% difference), and decrease in HDL-cholesterol (52.41% difference) (Table 4). Captopril mitigated only slightly the increases in triglyceride, total cholesterol, and LDL-cholesterol, and decrease in HDL-cholesterol mediated by L-NAME treatment (Table 4).

L-NAME treatment also increased the values of cardiac risk ratio (CRR) and atherogenic index (AI) (7.22 and 0.66, respectively) in hypertensive animals, compared to the normotensive control group (2.15 and 0.04, respectively) (*p* < 0.05) (Table 4). *A. digitata* doses 100 and 200 mg/kg prevented the development of these alterations (2.70 and 0.34 with 100 mg/kg and 3.49 and 0.35 with 200 mg/kg, *p* < 0.01) (Table 4). Although in a lesser extent compared to the effects of the extract, captopril also prevented the increment in CRR and AI values (2.73 and 0.08, respectively, *p* < 0.05) (Table 4).

### 3.3. Markers of Liver and Kidney Functions

Table 5 presents the changes in serum total protein, creatinine level, concentrations of sodium, potassium and chloride, and activities of ALAT and ASAT of animals treated daily and concomitantly with L-NAME and either the aqueous extract of *A. digitata* or captopril for 3 weeks.

#### 3.3.1. Liver Function

Hypertensive control animals displayed significant (*p* < 0.01) increases in serum total protein (79.10%), and ALAT (32.84%) and ASAT (53.99%) activities compared with the control group (Table 5). Treatment with captopril or extract doses 100 and 200 mg/kg significantly reduced (*p* < 0.001) ALAT and ASAT activities (27.87%, 30.69%, and 31.07%, and 57.42%, 47.23 %, and 49.69%, respectively) compared to hypertensive control group (Table 5). The increase in blood total protein observed in the hypertensive control group was mitigated significantly (*p* < 0.01) in the groups treated with extract dose 100 mg/kg (23.33% compared to the hypertensive control group), dose 200 mg/kg (26.67%), and captopril (25.83%) (Table 5).

#### 3.3.2. Kidney Function

Serum sodium and chloride concentrations and serum creatinine level were significantly increased, while serum potassium concentration was decreased, in hypertensive control animals compared to the normotensive control group (20.86%, *p* < 0.001, 0.63%, *p* < 0.05, and −46.55%, *p* < 0.001, respectively) (Table 5). Concomitant treatment with captopril or the extract significantly mitigated the L-NAME-induced increases in concentrations of sodium and chloride and in the level of creatinine, as well as the decrease in potassium concentration (Table 5).

### 3.4. Histopathological Changes in the Aorta, Heart, and Kidney

**Figure 2:**
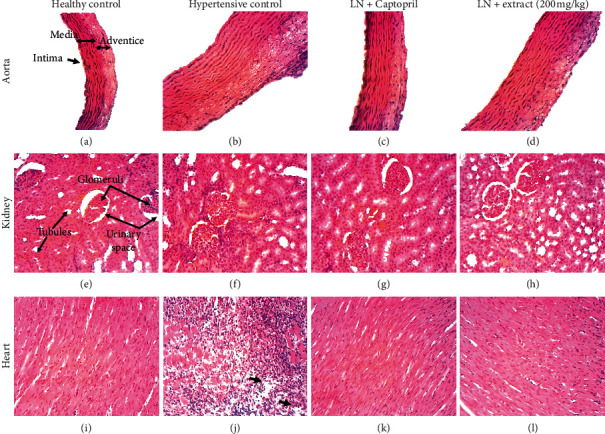
Aorta, heart, and kidney histopathology.

Figure 2 shows micrographs of H&E-stained sections of aortas, hearts, and kidneys of representative animals of the normotensive control group (Figures 2(a), 2(e), and 2(i)), of the hypertensive control group (Figures 2(b), 2(f), and 2(j)), and of animals treated concomitantly with L-NAME and either captopril (Figures 2(c), 2(g), and 2(k)) or dose 200 mg/kg of *A. digitata* extract (Figures 2(d), 2(h), and 2(l)). Overall, histopathological analyses of sections of the aorta revealed a marked thickening in the arterial walls leading to the reduction of blood vessel diameter (Figure 2(b)). Histopathological analyses of renal tissue of hypertensive control animals revealed tubular clarification, fusion of the glomeruli with the capsule, and focal peritubular and marked periglomerular infiltration of inflammatory cells were observed (Figure 2(f)). Histopathological analyses of sections of cardiac muscle tissue of hypertensive control animals revealed a marked infiltration of inflammatory cells, massive cell loss, congestion of blood vessels, and focal hemorrhagic lesions (Figure 2(j)). Treatment with captopril (Figures 2(c), 2(g), and 2(k)) or the aqueous extract of *A. digitata* (Figures 2(d), 2(h), and 2(l)) mitigated the histopathological changes induced by L-NAME treatment in the aorta, the heart, and the kidney.

Micrographs of H&E-stained section of aortas, hearts, and kidneys of representative animals of the normotensive control group (Figures 2(a), 2(e), and 2(i), respectively), of the hypertensive control group (Figures 2(b), 2(f), and 2(j), respectively), and of animals treated concomitantly with L-NAME (LN) and either captopril (Figures 2(c), 2(g), and 2(k), respectively) or dose 200 mg/kg of *Adansonia digitata* extract (Figures 2(d), 2(h), and 2(l), respectively). Note that unlike animals of the other groups, the hypertensive control animal displayed a marked (about 3-fold) increase in the thickness of the aorta wall (Figure 2(b)), an inflammation in the kidney revealed by a marked infiltration of inflammatory cells (Figure 2(f)), and both a massive infiltration of immune cells and cell loss (black arrows) in the heart (Figure 2(j); magnification, 100x).

## 4. Discussion

The results of the present study suggest that the aqueous extract of stem bark of *A. digitata* had preventive effects on the development of hypertension in L-NAME-induced hypertensive rats. Treatment of animals with L-NAME resulted in significant increases in heart rate and systolic, diastolic, and mean arterial blood pressures, as well as a reduction in weight gain in hypertensive control animals, which is in accordance with the available information on this model [30–32]. Concomitant treatment of animals with L-NAME and either the extract of *A. digitata* or the angiotensin-converting enzyme inhibitor captopril mitigated the blood pressure increase and prevented the reduction in weight gain observed in hypertensive control animals. L-NAME-induced dyslipidemia, which plays a pivotal role in the pathogenesis of hypertension in this model [33, 34], was not observed in animals concomitantly treated with L-NAME and either *A. digitata* extract or captopril in the present study, unlike hypertensive control animals that displayed marked decrease in blood HDL-cholesterol level and significant increases in blood levels of total cholesterol, LDL-cholesterol, and triglycerides. These observations suggest that, as captopril, the extract of *A. digitata* mitigated the development of L-NAME-induced hypertension in rats.

Moreover, treatment with either *A. digitata* extract or captopril also prevented hypertension-associated affections of organs typically observed in L-NAME-induced hypertensive animals, including kidney affection as indicated by the prevention of increases in creatinine and serum urea [35, 36] and liver affection, as indicated by the mitigation of the elevation of transaminase activity in the blood [37, 38]. In addition, hypertension can lead to abnormalities in cardiac structure [39, 40] and dysfunction in cardiac electrical activity [41, 42]. In this study, a significant increase in the relative weight of the left ventricle was observed in L-NAME-induced hypertensive control rats, but not in animals concomitantly treated with either captopril or the extract of *A. digitata*, further suggesting that *A. digitata* stem bark has antihypertensive properties. On the same hand, unlike in animals concomitantly treated with either captopril or the extract, increases in the magnitude of the QRS complex and in the duration of the QT and QRS intervals were observed in hypertensive control rats. In a previous report, L-NAME-treated rats displayed increases in RR interval and in the duration of the P wave, as well as an elevation in ST-segment, resulting in bradycardia [42, 43]. Our findings are in agreement with this report, as prolongation of QT interval may indicate ventricular arrhythmia and QRS interval lengthening a slowing down of the frequency of heart contractions [44–46].

The histopathological findings confirmed the biochemical and the electrophysiological observations. Histopathological changes typically observed in L-NAME-induced hypertensive rats, such as increased monocyte and platelet adhesion, thickening of the vascular wall, aggregation of inflammatory cells in organs [47, 48], were less marked or absent in animals treated with either *A. digitata* extract or captopril. Altogether, these findings suggest that *A. digitata* extract has antihypertensive properties in L-NAME-induced hypertensive rats.

The mitigation of decreases in releases of endothelial NO observed following treatment with *A. digitata* in this study suggests that the extract mediated its antihypertensive activity at least partly by improving NO bioavailability [7–10]. In addition, in this study, *A. digitata* extract prevented hypertension-like impairment of antioxidant defenses indicated by marked decreases in nitrite levels, reduced glutathione levels, and catalase activity, and increases in levels of lipid peroxidation indicator malondialdehyde in the heart, aorta, and kidneys in hypertensive control rats [49–51]. These observations suggest that *A. digitata* extract has antioxidant properties. Such properties may contribute to the antihypertensive effects of the extract, considering the pivotal role of oxidative stress in the development of hypertension in L-NAME-induced hypertensive rats [5, 6, 49].

## 5. Conclusions

We assessed the effects of the aqueous extract of *A. digitata* stem bark on the development of hypertension in L-NAME-induced hypertension rat models. The extract prevented or mitigated the hypertension-like changes in hemodynamic and electrocardiographic parameters, serum lipid profile, and oxidative stress markers, as well as aorta, heart, and kidney histopathological signs. Altogether, these findings suggest the aqueous extract of stem bark *A. digitata* has antihypertensive and antioxidant properties, and justify its use in folk medicine against cardiovascular disorders.

## Figures and Tables

**Table 1:** Body, heart, and kidneys weights after 3 weeks of treatment.

Parameters	Normotensive control	Hypertensive ctrl	LN + captopril	LN + AD100	LN + AD200
Body weight week 3 (g)	229.95 ± 2.02	209.37 ± 0.80^c^	219.10 ± 1.68^*δ*^	206.50 ± 1.38	216.35 ± 0.77^*δ*^
Body W gain (% baseline)	26.11 ± 2.91	11.77 ± 3.48^a^	12.79 ± 1.79	22.85 ± 1.55^*α*^	14.95 ± 6.97^*δ*^
Kidney W (g/100 g BW)	0.59 ± 0.05	0.79 ± 0.05^c^	0.60 ± 0.01^*δ*^	0.56 ± 0.01^*δ*^	0.61 ± 0.01^*δ*^
Heart W (g/100 g BW)	0.32 ± 0.02	0.37 ± 0.02^c^	0.31 ± 0.03^*δ*^	0.29 ± 0.01^*β*^	0.34 ± 0.01
Left ventricle W (g/100 g)	0.13 ± 0.01	0.18 ± 0.01^a^	0.16 ± 0.01	0.14 ± 0.01^*δ*^	0.16 ± 0.01

Data are mean ± SEM (*N* = 6 per group). ANOVA + the Tukey test: ^c^*p* < 0.05 and ^a^*p* < 0.001 vs. the normotensive control group; ^*δ*^*p* < 0.05, ^*β*^*p* < 0.01, and ^*α*^*p* < 0.001 vs. the hypertensive control group. AD100/AD200 = extract doses 100 or 200 mg/kg. LN = N-NAME.

**Table 2:** Effects of *A. digitata* extract on blood pressure and heart rate of hypertensive rats.

	SBP (mm Hg)	DBP (mm Hg)	MABP (mm Hg)	HR (BPM)
Normotensive control	113.24 ± 2.85	81.63 ± 3.26	92.17 ± 3.11	345.61 ± 4.65
Hypertensive control	189.49 ± 3.85^a^	147.26 ± 5.40^a^	161.34 ± 4.79^a^	390.28 ± 16.45^c^
LN + captopril	147.18 ± 5.82^*α*^	118.65 ± 5.35^*α*^	128.16 ± 5.32^*α*^	349.98 ± 8.27
LN + AD100	172.64 ± 1.61^*α*^	135.77 ± 1.50^*β*^	148.06 ± 1.40^*α*^	349.48 ± 13.16
LN + AD200	149.07 ± 7.86^*α*^	115.10 ± 6.73^*α*^	126.42 ± 7.08^*α*^	341.04 ± 19.20^*α*^

Data are mean ± SEM (*N* = 6 per group). ANOVA + the Tukey test: ^c^*p* < 0.05 and ^a^*p* < 0.001 vs. the normotensive control group; ^*δ*^*p* < 0.05, ^*β*^*p* < 0.01, and ^*α*^*p* < 0.001 vs. the hypertensive control group. AD100/AD200 = extract doses 100 or 200 mg/kg. DBP = diastolic arterial blood pressure. HR = heart rate. LN = N-NAME. MABP = mean arterial blood pressure. SBP = systolic arterial blood pressure.

**Table 3:** Effects of *A. digitata* extract on the electrocardiogram of hypertensive rats.

ECG Parameter	Time (ms)						Magnitude (mV)	
	P-R Int	QRS Int	Q-T Int	S-T Int	S-T Seg	R-R Int	P wave	QRS complex
Normotensive ctrl	43.3 ± 0.3	22.0 ± 1.0	66.0 ± 3.9	35.0 ± 0.5	5.0 ± 0.1	148.3 ± 0.4	0.09 ± 0.01	0.58 ± 0.01
Hypertensive ctrl	42.7 ± 0.6	37.7 ± 0.6^c^	85.7 ± 0.6^b^	39.3 ± 0.3	8.7 ± 0.3	159.3 ± 0.3	0.08 ± 0.01	0.61 ± 0.01
LN + captopril	45.7 ± 0.3	19.7 ± 0.8^*δ*^	62.0 ± 0.3^*β*^	40.3 ± 1.2	7.0 ± 0.5	168.8 ± 0.6	0.08 ± 0.01	0.48 ± 0.02
LN + AD100	49.3 ± 0.6^*α*^	30.3 ± 0.8	65.3 ± 1.0^*β*^	40.3 ± 0.8	6.6 ± 0.3	169.7 ± 0.8	0.12 ± 0.01	0.73 ± 0.02
LN + AD200	49.3 ± 0.6^*α*^	21.0 ± 0.0^*δ*^	65.6 ± 1.5^*β*^	46.0 ± 0.9	7.6 ± 0.3	156.0 ± 1.0	0.11 ± 0.01	0.43 ± 0.02

Data are mean ± SEM (*N* = 6 per group). ANOVA + the Tukey test: ^b^*p* < 0.01 vs. the normotensive control group; ^*δ*^*p* < 0.05, ^*β*^*p* < 0.01, and ^*α*^*p* < 0.001 vs. the hypertensive control group. AD100/AD200 = extract doses 100 or 200 mg/kg. Int = interval. LN = N-NAME. Seg = segment.

**Table 4:** Effects of *A. digitata* extract on blood lipid profile of hypertensive rats.

	Normotensive control	Hypertensive ctrl	LN + captopril	LN + AD100	LN + AD200
Total cholesterol (mg/dL)	65.24 ± 1.28	80.47 ± 1.61^b^	66.02 ± 1.70^*α*^	69.90 ± 2.14	59.42 ± 1.85^*δ*^
Triglycerides (mg/dL)	33.29 ± 1.57	51.07 ± 0.86^c^	28.98 ± 1.58^*δ*^	40.82 ± 6.69	37.94 ± 3.26^*β*^
HDL-cholesterol (mg/dL)	30.28 ± 3.62	11.15 ± 0.64^b^	24.18 ± 3.27^*δ*^	18.87 ± 0.50	17.02 ± 2.69
LDL-cholesterol (mg/dL)	28.21 ± 4.47	45.52 ± 3.65^b^	36.04 ± 3.31	42.87 ± 1.58	34.81 ± 2.81
Cardiac risk ratio	2.15	7.22^a^	2.73^*β*^	3.70^*δ*^	3.49^*δ*^
Atherogenic index	0.04	0.66	0.08	0.34	0.35

Data are mean ± SEM (*N* = 6 per group). ANOVA + the Tukey test: ^c^*p* < 0.05, ^b^*p* < 0.01, and ^a^*p* < 0.001 vs. the normotensive control group; ^*δ*^*p* < 0.05, ^*β*^*p* < 0.01, and ^*α*^*p* < 0.001 vs. the hypertensive control group. AD100/AD200 = extract doses 100 or 200 mg/kg. LN = N-NAME.

**Table 5:** Effects of *A. digitata* on blood indicators of liver and kidney functions in hypertensive rats.

	Normotensive control	Hypertensive control	LN + captopril	LN + AD100	LN + AD200
ASAT (UI/L)	116.7 ± 6.13	179.7 ± 11.52^a^	76.5 ± 7.31^*α*^	94.8 ± 9.74^*α*^	106.6 ± 7.22^*α*^
ALAT (UI/L)	41.6 ± 1.36	55.3 ± 4.37^b^	39.9 ± 2.09^*β*^	38.4 ± 1.81^*β*^	38.1 ± 1.64^*β*^
Total protein (mg/dL)	1.3 ± 0.08	2.4 ± 0.11^a^	1.8 ± 0.52^*β*^	1.8 ± 0.05^*β*^	1.8 ± 0.07^*β*^
Creatinine (mg/dL)	4.8 ± 1.68	15.6 ± 1.29^a^	4.9 ± 0.28^*α*^	6.5 ± 0.28^*α*^	3.5 ± 3.12^*α*^
Sodium (mEq/L)	115.4 ± 1.89	139.4 ± 8.03^b^	116.8 ± 1.63^*α*^	100.6 ± 2.97^*α*^	86.6 ± 7.38^*α*^
Potassium (mEq/L)	10.9 ± 0.14	6.1 ± 0.06^b^	8.8 ± 0.05^*β*^	11.6 ± 0.36^*α*^	15.2 ± 0.59^*α*^
Chloride (mEq/L)	87.6 ± 1.23	93.0 ± 0.91^c^	87.9 ± 0.30^*δ*^	80.3 ± 0.21^*α*^	86.0 ± 0.97^*β*^

Data are mean ± SEM (*N* = 6 per group). ANOVA + the Tukey test: ^b^*p* < 0.01 and ^a^*p* < 0.001 vs. the normotensive control group; ^*δ*^*p* < 0.05, ^*β*^*p* < 0.01, and ^*α*^*p* < 0.001 vs. the hypertensive control group. AD100/AD200 = extract doses 100 or 200 mg/kg. LN = N-NAME.

## Data Availability

The datasets supporting the conclusions of this article are presented in this main paper. Plant materials used in this study have been identified at the Cameroon National Herbarium where voucher specimens are deposited.

## Supplementary Materials

Supplementary Materials The supplementary file includes the raw data of the article.

## References

[1] Tian D., Ling S., Chen G. (2011). Hypertensive nephropathy treatment by heart-protecting musk pill: a study of anti-inflammatory therapy for target organ damage of hypertension. *International Journal of General Medicine*.

[2] Binda D., Nicod L., Viollon-Abadie C. (2001). Strain difference (WKY, SPRD) in the hepatic antioxidant status in rat and effect of hypertension (SHR, DOCA). Ex vivo and in vitro data. *Molecular and Cellular Biochemistry*.

[3] Opie L. H., Seedat Y. K. (2005). Hypertension in sub-Saharan African populations. *Circulation*.

[4] Kingue S., Ngoe C. N., Menanga A. P. (2015). Prevalence and risk factors of hypertension in urban areas of Cameroon: a nationwide population-based cross-sectional study. *The Journal of Clinical Hypertension*.

[5] Baradaran A., Nasri H., Rafieian-Kopaei M. (2014). Oxidative stress and hypertension: possibility of hypertension therapy with antioxidants. *Journal of Research in Medical Sciences: The Official Journal of Isfahan University of Medical Sciences*.

[6] Sinha N., Dabla P. (2015). Oxidative stress and antioxidants in hypertension-A current review. *Current Hypertension Reviews*.

[7] Moncada S., Palmer R. M., Higgs E. A (1991). Nitric oxide: physiology, pathophysiology, and pharmacology. *Pharmacological Reviews*.

[8] Shin W., Cuong T. D., Lee J. H. (2011). Arginase inhibition by ethylacetate extract ofCaesalpinia sappanLignum contributes to activation of endothelial nitric oxide synthase. *The Korean Journal of Physiology and Pharmacology*.

[9] World Health Organization (2013). *A Global Brief on Hypertension: Silent Killer, Global Public Health Crisis*.

[10] Zafar K., Mushtaq A. (2015). Assessing the primary causes of hypertension in Khyber Pakhtunkhunwa, Pakistan. *Journal of Biology and Life Science*.

[11] Kamatou G. P. P., Vermaak I., Viljoen A. M. (2011). An updated review of *Adansonia digitata*: a commercially important African tree. *South African Journal of Botany*.

[12] Ibrahima C., Didier M., Max R., Pascal D., Benjamin Y., Renaud B. (2013). Biochemical and nutritional properties of baobab pulp from endemic species of Madagascar and the African mainland. *African Journal of Agricultural Research*.

[13] Li X. N., Sun J., Shi H. (2017). Profiling hydroxycinnamic acid glycosides, iridoid glycosides, and phenylethanoid glycosides in baobab fruit pulp (*Adansonia digitata*). *Food Research International*.

[14] Mohammad Y. G., Hauwa’u Y. B. (2013). Hypoglycemic activity of methanolic fruit pulp extract of *A. digitata* on blood glucose levels of alloxan induced diabetic rats. *International Journal of Animal and Veterinary Advances*.

[15] Rahul J., Jain M. K., Singh S. P., Kamal R. K., Anuradha N., Mrityunjay S. K. (2015). *Adansonia digitata* L. (baobab): a review of traditional information and taxonomic description. *Asian Pacific Journal of Tropical Biomedicine*.

[16] De Caluwé E., Halamová K., Van Damme P. (2010). *Adansonia digitata* L.-a review of traditional uses. Phytochemistry Pharmacology. *Afrika Focus*.

[17] Adeoye A. O., Bewaji C. O. (2018). Chemopreventive and remediation effect of *Adansonia digitata* L . Baobab (Bombacaceae) stem bark extracts in mouse model malaria. *Journal of Ethnopharmacology*.

[18] Shehu A., Magaji M. G., Yau J., Abubakar A. (2017). Ethnobotanical survey of medicinal plants used for the management of depression by Hausa tribes of Kaduna state, Nigeria. *Journal of Medicinal Plants Research*.

[19] Shehu A., Magaji M. G., Magaji M., Mahmud B., Ahmed A. (2018). Antidepressant effect of methanol stem bark extract of *Adansonia digitata* L. (Malvaceae) in mice. *Tropical Journal of Natural Product Research*.

[20] Mahmoud E. K., Ghoneim A. M. (2016). Effect of polluted water on soil and plant contamination by heavy metals in El-Mahla El-Kobra, Egypt. *Solid Earth*.

[21] Alhassan A. J., Muhammad I. U., Jarumi A. M., Wudil A. M. (2016). Evaluation of anti-hyperlipidemic potentials of aqueous fruit pulp extract of *adensonia digitata* in experimental rats. *European Scientific Journal*.

[22] Bartels H., Böhmer M., Heierli C. (1972). Serum kreatininbestimmung ohne enteiweissen. *Clinica Chimica Acta*.

[23] Gornall A. G., Bradwill C. J., David M. M. (1949). Determination of serum proteins by the mean of the biuret reactions. *Journal of Biological Chemistry*.

[24] Reitman S., Frankel S. (1957). A colorimetric method for the determination of serum glutamic oxalacetic and glutamic pyruvic transaminases. *American Journal of Clinical Pathology*.

[25] Wakayashi I., Kobaba W. R. (2002). Effet de l’âge sur le rapport entre le boire et les rapports arthérosclérotiques. *Gerontology*.

[26] Ellman G. L. (1959). Tissue sulfhydryl groups. *Archives of Biochemistry and Biophysics*.

[27] Sinha A. K. (1972). Colorimetric assay of catalase. *Analytical Biochemistry*.

[28] Slack P. T. (1987). *Analytical Methods Manual*.

[29] Wilbur K. M., Bernheim F., Shapiro O. W. (1949). Determination of lipid peroxidation. *Archives of Biochemistry and Biophysics*.

[30] Bilanda D. C., Dimo T., Dzeufiet Djomeni P. D. (2010). Antihypertensive and antioxidant effects of Allanblackia floribunda Oliv. (Clusiaceae) aqueous extract in alcohol- and sucrose-induced hypertensive rats. *Journal of Ethnopharmacology*.

[31] Czech D. A., Kazel M. R., Harris J. (2003). A nitric oxide synthase inhibitor, NG-nitro-l-arginine methyl ester, attenuates lipoprivic feeding in mice. *Physiology & Behavior*.

[32] Farr S. A., Banks W. A., Kumar V. B., Morley J. E. (2005). Orexin-A-induced feeding is dependent on nitric oxide. *Peptides*.

[33] Saravanakumar M., Raja B. (2012). Effect of veratric acid on the cardiovascular risk of L-NAME-induced hypertensive rats. *Journal of Cardiovascular Pharmacology*.

[34] Tsague M. V., Fokunang N. C., Tembe A. E. (2016). Hydroethanolic extract of *Eribroma oblongum* (malvaceae) stem bark. *Journal of Diseases and Medicinal Plants*.

[35] Lameire N., Vanbiesen W., Vanholder R. (2005). Acute renal failure. *The Lancet*.

[36] Tsuchiya K., Tomita S., Ishizawa K. (2010). Dietary nitrite ameliorates renal injury in L-NAME-induced hypertensive rats. *Nitric Oxide*.

[37] Prahalathan P., Kumar S., Raja B. (2012). Effect of morin, a flavonoid against DOCA-salt hypertensive rats: a dose dependent study. *Asian Pacific Journal of Tropical Biomedicine*.

[38] Li X., Luo Y., Wang L. (2010). Acute and subacute toxicity of ethanol extracts from Salvia przewalskii Maxim in rodents. *Journal of Ethnopharmacology*.

[39] Miguel-Carrasco J. L., Monserrat M. T., Mate A., Vázquez C. M. (2010). Comparative effects of captopril and L-carnitine on blood pressure and antioxidant enzyme gene expression in the heart of spontaneously hypertensive rats. *European Journal of Pharmacology*.

[40] Cuspidi C., Rescaldani M., Sala C., Negri F., Grassi G., Mancia G. (2012). Prevalence of electrocardiographic left ventricular hypertrophy in human hypertension. *Journal of Hypertension*.

[41] Mozos I., Caraba A. (2015). Electrocardiographic predictors of cardiovascular mortality. *Disease Markers*.

[42] Mansur P. H., Cury L. K., Destro-Filho J. B. (2006). Analysis of electrocardiographic recordings associated with acute myocardial infarction. *Arquivos Brasileiros de Cardiologia*.

[43] El-Mosallamy A. E. M. K., Sleem A. A., Abdel-Salam O. M. E., Shaffie N., Kenawy S. A. (2012). Antihypertensive and cardioprotective effects of pumpkin seed oil. *Journal of Medicinal Food*.

[44] Feldman J., Goldwasser G. P. (2004). Electrocardiogram: recommendations for interpretation. *Chemical Society Reviews*.

[45] Verweij N., Leach I. M., van den Boogaard M. (2014). Genetic determinants of P wave duration and PR segment. *Circulation: Cardiovascular Genetics*.

[46] Bjerregaard P., Nallapaneni H., Gussak I. (2010). Short QT interval in clinical practice. *Journal of Electrocardiology*.

[47] Dolores P. M., Paola R., Noemí M. (2018). Beneficial effects of different flavonoids on vascular and renal function in LN hypertensive rats. *Nutrients*.

[48] Almeida Rezende B., Pereira A. C., Cortes S. F., Lemos V. S. (2016). Vascular effects of flavonoids. *Current Medicinal Chemistry*.

[49] Rodrigo R., Prat H., Passalacqua W., Araya J., Guichard C., Bächler J. P. (2007). Relationship between oxidative stress and essential hypertension. *Hypertension Research*.

[50] Nayeemunisa, Kumda M. R. (2003). Cardioprotective effects of *Cichorium intybus* in ageing myocardium of albino rats. *Current Science*.

[51] Ahmad A., Singhal U., Hossain M. M., Islam N., Rizvi I. (2013). The role of the endogenous antioxidant enzymes and malondialdehyde in essential hypertension. *Journal of Clinical and Diagnostic Research*.

